# Translation and Validation of Nursing Students’ Knowledge and Attitudes of Lesbian, Gay, Bisexual, Transgender Health Concerns Survey in the Greek Language

**DOI:** 10.3390/healthcare10122547

**Published:** 2022-12-15

**Authors:** Evangelos C. Fradelos, James Montegrico, Judith Cornelius, Vissarion Bakalis, Maria Malliarou, Ioanna V. Papathanasiou, Georgia Fasoi, Martha Kelesi, Evridiki Kaba, Victoria Alikari

**Affiliations:** 1 Faculty of Nursing, University of Thessaly, 41500 Larissa, Greece; 2 School of Nursing, The University of North Carolina at Charlotte, Charlotte, NC 28213, USA; 3 Department of Nursing, University of West Attica, 12243 Athens, Greece

**Keywords:** nursing students, LGBT care, scale, sexuality, validation

## Abstract

Nursing students have limited knowledge of providing quality care to lesbian, gay, bisexual, and transgender individuals. The aim of this study was to explore the psychometric properties of the Nursing Students’ Knowledge of and Attitudes toward Lesbian, Gay, Bisexual, and Transgender (LGBT) Health Concerns (NKALH) survey as well as to examine whether sociodemographic variables may affect attitudes toward LGBT individuals. In this cross-sectional study which was carried out from January–March 2022, 258 nursing students from two Greek universities completed the NKALH. For survey translation, the procedure of forward and backward translation was followed. Construct validity, reliability, and internal consistency were explored via confirmatory factor analysis, the test–retest reliability method, and Cronbach’s alpha index. The correlation between the dimensions of the attitude subscale was used to explore convergent validity. Analyses were carried out with the Statistical Package for the Social Sciences (SPSS), version 26.0. The level of significance was set at <0.05. Confirmatory factor analysis confirmed that the attitude subscale consists of three dimensions (Comfortable, Responsibility, Willingness to Care). The Pearson test (r) revealed strong correlations between two measurements (Comfortable r = 0.932, *p* < 0.001, Responsibility r = 0.938, *p* < 0.001, and Willingness to Care r = 0.915, *p* < 0.001), indicating good reliability. The Cronbach’s alpha index of the total scale was 0.783, highlighting its good internal consistency. Bivariate analysis revealed that sexual orientation, religion, and age are related to knowledge and attitudes toward LGBT individuals. The NKALH survey is a valid and reliable tool to measure the knowledge and attitudes of Greek nursing students on the health concerns of LGBT individuals.

## 1. Introduction

Lesbian, gay, bisexual, and transgender (LGBT) individuals experience prejudice and stigma on a daily basis even in their encounters with healthcare professionals [1]. According to the World Health Organization, these individuals are less likely to use healthcare services due to this structural stigma and discrimination. Moreover, LGBT individuals will deny health issues due to discriminatory attitudes and inappropriate pathologizing (considering sexual preferences, desires, and behaviors as disorders) in healthcare settings and thus have poor physical and mental health outcomes [2,3]. In addition, this population experiences unique healthcare needs when compared to the general population. For example, anal cancer, breast cancer, substance abuse, cardiovascular diseases, and mental health issues occur more frequently among LGBT individuals when compared to heterosexuals [4].

Although LGBT individuals are an important proportion of the population globally [5], there is still a gap in knowledge regarding the healthcare issues of this population among healthcare professionals, including nursing students [6]. A cross-sectional study in the United States among 268 nurses revealed that clinical nurses did not have any education or training on LGBT health issues. This knowledge gap may lead to discomfort when delivering care to LGBT individuals and thus adversely affect the quality of care that they receive. The authors of the study conclude that LGBT healthcare education needs to be integrated into nursing curriculums, programs, and continuing education [6]. Similarly, Cornelius and Carrick [7] compared a sample of registered nurse-to-bachelor of science of nursing students to other undergraduate and graduate nursing students and reported that the majority of the former students had significant LGBT healthcare knowledge and more positive attitudes toward caring for LGBT patients when compared to the undergraduate and graduate students. The authors recommended that supplementary teaching strategies are needed in order for students to feel comfortable in providing care to LGBT individuals [7]. 

In a recent study with nursing students in Turkey, male students reported having more discriminating attitudes towards LGBT individuals when compared to female students [8]. Various demographic factors (gender, race, and if they know an LGBT person) can be associated with attitudes that nursing students have toward LGBT individuals and their comfort with caring for them [8,9,10]. Research findings indicate that further exploration of this topic is needed [7,8,9,10]. 

Similar to other countries, in Greece LGBT individuals face discrimination and negative attitudes [11]. While these attitudes toward LGBT individuals have been examined in the general population [12], knowledge and attitudes toward caring for LGBT patients have not been examined among nursing students in Greece. Thus, the purpose of this study is to validate a scale that examines knowledge and attitudes toward LGBT individuals in the Greek language. Also, the psychometric properties of the Greek version could be compared to those of the original version, thus covering a gap in the literature. A secondary aim of this study is to examine whether sociodemographic variables may affect attitudes toward LGBT individuals in a sample of Greek nursing students.

## 2. Materials and Methods

This is a cross-sectional study with nursing students selected from two universities (the University of Thessaly and the University of West Attica). Among the 370 students that were invited to participate, 260 provided consent (response rate 70%). The inclusion criteria were (i) being nursing students and (ii) attending an undergraduate program. The questionnaires were distributed online from January–March 2022.

### 2.1. Instrument

Students completed the Nursing Students’ Knowledge of and Attitudes toward LGBT Health Concerns (NKALH) survey [7]. The scale was developed in 2015 by Cornelius and Carrick [7] and consists of 70 items. The scale has two parts. The first part consists of 37 questions regarding the knowledge of nursing students on LGBT healthcare issues (e.g., substance and tobacco use, cardiovascular risk, cancer risk, sex reassignment surgery, acts of violence, HIV/AIDS, sexually transmitted diseases, and immunizations). Each correct answer is graded with a 1. Incorrect answers as well as “don’t know” answers are graded with a 0. The second part has 25 questions that explore students’ attitudes toward providing healthcare to LGBT people (nursing responsibilities, comfort and willingness to care, and views about same-sex intimate relationships). Participants responded using a five-point Likert scale (1 = strongly agree to 5 = strongly disagree). The higher the scores, the more positive the self-reported attitudes and comfort in providing care for LGBT patients [7]. Sociodemographic data of nursing students were also recorded. 

### 2.2. The Translation and Cultural Adaptation Process

To translate the scale, the guidelines of the International Society for Pharmacoeconomics and Outcomes Research (ISPOR) (Task Force for Translation and Cultural Adaptation) [13] were followed. In the first stage, the procedure of forward translation was applied. Two Greek, bilingual nurses, fully conversant with English culture, translated the scale from English (source language) to Greek (target language). The principal investigator of this study collated the two Greek translations, and a different Greek translation of the scale was obtained (first reconciliation version). In the second stage, the procedure of backward translation was followed. An independent, bilingual translator translated the Greek version into the English language without having read the original English version. Thus, the second reconciliation version emerged.

For cultural adaptation, at least 10 participants are required [13]. The cognitive interview process followed, in which the nursing students stated whether there were any unclear and difficult-to-understand points. In this review, the students provided different wordings without, however, altering the meanings of the questions (cognitive account review). Researchers incorporated their suggestions into the second reconciliation version, thus generating the final Greek version of the NKALH. Students then completed a general impressions tool in which students indicated whether there were unfamiliar words or difficult words present in the survey. The majority of the students (*n* = 9) stated that there were no unclear points. 

### 2.3. Reliability

The test–retest method was applied to explore the test–retest reliability of the NKALH. In particular, 30 nursing students completed the questionnaire at baseline and two weeks later. This interval is interposed so that the individuals do not recall their answers [14]. Cronbach’s alpha index was used to test the internal consistency reliability of the NKALH.

### 2.4. Data Analysis

Analyses were performed with the Statistical Package for the Social Sciences (SPSS), version 26.0 (IBM Corp., Armonk, NY, USA), and JASP v.0.14.1.0 The statistical significance was set at <0.05. To describe quantitative variables, mean values and standard deviations were applied. Absolute and relative frequencies were used to describe qualitative variables. To assess the test–retest reliability between the two measurements, a paired sample *t*-test was used. To assess the internal consistency reliability of the scale, Cronbach’s alpha index was applied (accepted values > 0.70) [14]. The construct validity and the three-factor structure of the NKALH were tested through confirmatory factor analysis. Specifically, the following fit statistics were used to explore the model’s global adjustment: the Tucker–Lewis index (TLI), the root-mean-square error of approximation (RMSEA), and the comparative fit index (CFI). Values of CFI, TLI ≥ 0.90, and RMSEA ≤ 0.10 indicate an acceptable fit [14,15]. Correlations between the item scores and the total score of each subscale were applied. Values of the correlation coefficient (r) from 0.1–0.3 indicate low correlation, from 0.31–0.5 moderate, and >0.5 high correlation [16]. 

### 2.5. Ethics

To conduct the study, licenses were secured from the Research Ethics Committee IRB of the Universities of Thessaly and West Attica (approval number 63/30 August 2021). The students were informed in writing about the scope of the work, the anonymity and confidentiality of the data, that the data would not be shared with anyone, and their right to absent themselves, if they so desired. All the nursing students signed a written informed consent form.

## 3. Results

The students’ sociodemographic characteristics are presented in Table 1. The majority (81.4%) were female and identified as heterosexuals (80.6%). The mean age was 23.5 years old (SD ± 7.8).

### 3.1. Descriptive Statistics

The descriptive statistics for the three scales as well as for the score of the knowledge quiz of the study are presented in Table 2. As noted, the total score of the attitudes scales and three individual subscales’ mean scores are above 2, and thus we can say that nursing students have positive attitudes toward caring for LGBT patients. Moreover, the mean of knowledge was 16.49, meaning that the majority of students correctly answered less than half of the knowledge questions regarding LGBT-specific health concerns. In addition, Cronbach’s alpha index was found to be above 0.70 in the total scale as well as in all subscales, indicating that the scale has good internal consistency.

### 3.2. Construct Validity of the NKALH Survey

Confirmatory factor analyses (CFA) were performed in the second part of the NΚALH (25 questions), which assessed students’ attitudes toward providing healthcare to LGBT people. CFA was performed to explore the structural validity of the NKALH. The model of this study was equivalent to the original factorial structure of the NKALH survey as recommended by the constructors. This model presented a reasonably good fit to the data. TLI was 0.847 (>0.8 and <0.9), CFI was 0.873 close to 0.9, and RMSEA was 0.086 and lower than 0.10. In general, the CFA verified the structure of the three dimensions of the NKALH survey. Moreover, a positive correlation between all the subscales of the NKALH (*p* < 0.001) was shown, as the highest value of r was found to be 0.907, and the lowest was 0.55. This result reflects that indeed all three factors are parts of the same construct (Table 3).

### 3.3. Test–Retest Reliability

Thirty participants completed the NKALH survey at baseline and two weeks later. From the total 30 students, 76.7% (23) were female, 80.2% were heterosexual, and 40% (12) were in the second year of their studies. The mean age was 22.03 ± 7.8. Between the two measurements, no significant differences were observed. This indicates the test–retest reliability of the scale. No significant differences were observed between the two measurements according to the paired sample *t*-test. Also, between the two measurements, strong correlations emerged, as the Pearson correlation coefficient r for Comfortable was r = 0.932 (*p* < 0.001), for Responsibility it was r = 0.938 (*p* < 0.001), and for the subscale Willingness for Care it was r = 0.915 (*p* < 0.001).

### 3.4. Bivariate Analysis

In order to identify sociodemographic characteristics that are associated with students’ knowledge and attitudes toward caring for LGBT patients, bivariate analysis was performed. According to the analysis, we observed that nursing students are more willing to care for LGBT patients than nursing assistants that are attending a university for a degree upgrade—4.38 vs. 3.98, t(258) = 2.768, *p* = 0.008, 95% CI [−1,435–3,255]. Students who have a close friend or relative who identifies as an LGBT individual exhibited more positive attitudes and reported greater Knowledge of LGBT health concerns when compared to other students (14.3 vs. 16.7, t(258) = −2.269, *p* = 0.001, 95% CI [−4,568–0,323]. For the Comfortable dimension, the results were 3.70 vs. 3.98, t(258) = −2.459, *p* = 0.019, 95% CI [−0,45364–0,10923]. For Responsibility they were 3.58 vs. 3.85, t(258) = −2.225, *p* = 0.027, 95% CI [−0,50695–0,03088]. For Willingness to Care, they were 3.86 vs. 4.41, t(258) = −4.046, *p* <0.001), 95% CI [−0,80742–0,27877].

According to one-way ANOVA, there was a statistically significant difference in knowledge of LGBT health concerns in the sexual orientation groups. F(3.252) = 2.826, *p* = 0.037). The Bonferroni test for multiple comparisons found that the mean Knowledge score on LGBT health concerns was significantly different between heterosexuals and homosexuals (*p* = 0.006). A similar statistically significant difference in responsibility to care between sexual orientation groups was observed: F(3.252) = 4.726, *p* = 0.003. The Bonferroni test for multiple comparisons found that the mean value of knowledge on LGBT health concerns was significantly different between heterosexuals and homosexuals (*p* = 0.004). Finally, there were statistically significant differences in knowledge and attitudes among the different religious groups.

Regarding the Knowledge dimension of LGBT health concerns, the Bonferroni Test for multiple comparisons found that the mean value of Knowledge on LGBT health concerns was significantly different between Christians and atheists/non-religious people when compared to Protestants and Muslims [F(2.256) = 3.296, *p* = 0.039]. The Bonferroni Test for multiple comparisons found that the mean value of Knowledge on LGBT health among. Muslims and Protestants was lower when compared to Christians and atheists/non-religious people. A similar finding was reported with the Comfortable subscale [F(2.215) = 5.887, *p* = 0.003], where the mean value of Comfortable was significantly different between Christians and atheists/non-religious participants when compared to Christians, Muslims, and Protestants, who scored lower than atheists (*p* = 0.001). Regarding the scale of Responsibility, statistical differences between the aforementioned groups were observed [F(2.256) = 7.372, *p* = 0.001]. More atheists and non-religious participants reported more positive attitudes when compared to Christians and Muslims or Protestants (*p* = 0.002). In addition, Christians had also more positive attitudes than other religious affiliations (*p* = 0.001). The same observations applied in the Willingness to Care subscale [F(2.256) = 13.235, *p* = 0.001)]. According to the Bonferroni test for multiple comparisons, atheists/non-religious participants had higher mean scores than Christians (*p* = 0.002) and Muslims or Protestants (*p* = 0.001), and Christians had higher mean scores on Willingness to Care when compared to Muslims and Protestants (*p* = 0.001). Finally, age was negatively correlated with the Comfortable (r = −0.152, *p* = 0.014), Responsibility (r = −0.210, *p* = 0.001), and Willingness to Care (r = −0.167, *p* = 0.007) scales, indicating that older participants have negative attitudes towards LGBT care.

## 4. Discussion

This study was conducted in two Departments of Nursing aiming to study the reliability and validity of the NKALH survey. This is the first time NKALH survey was used among Greek nursing students. The study is of great importance, as comprehension and understanding of students’ knowledge of and attitudes toward LGBT patients can help the development of a more comprehensive curriculum for nursing students in a way that contributes to more adequate care for LGBT patients [17].

According to the results, the Greek version of the NKALH survey is a validated and reliable tool. Because the psychometric properties of the NKALH survey have not been tested in other cultures, the reliability and validity results of the present study can only be compared with those of the scale construction study. As far as the internal consistency of the NKALH survey is concerned, the total Cronbach’s alpha index was found to be 0.783, indicating good internal consistency. Comparing this result to the total Cronbach’s alpha value of the original version, which was 0.77, we can say that it is rated at similar levels [7,18]. In this study, the subscale Comfortable had the lowest Cronbach’s alpha (0.701), while the subscale Willingness to Care had the highest one (0.816). In the study by Cornelius [18], the highest Cronbach’s alpha was 0.85 for the dimension of “Comfort in providing care”.

Concerning the survey’s structural validity, our results confirmed the three-factor model’s improvement on the original version of the survey [7]. The concepts of comfort with providing care, willingness, and a sense of responsibility are common both in the current study and previous studies [19] in which authors highlight that nursing students feel discomfort towards LGBT patients due to the lack of knowledge regarding the health issues of these persons. 

Consistent with the literature [7,20], nursing students have a lack of knowledge regarding LGBT-specific health concerns. The level of knowledge is low even among nurses [19]. This result should not be surprising, as sexual education is not fully developed in Greece. Together with other studies [7,21], this study emphasizes the need for increased knowledge and additional competencies, as the higher the level of knowledge on LGBT health issues, the higher the level of willingness and comfort toward LGBT patients [22]. In this study, the Greek nursing students had positive attitudes toward caring for LGBT patients. This finding is consistent with research among students in the healthcare sciences [23]. In another study in Greece among 548 undergraduate students of different healthcare departments, students had fewer positive attitudes towards LGBT persons compared to other professions [24]. Lim and Hsi [25] in a systematic review reported mixed findings regarding nursing students’ attitudes toward LGBT patients. According to this review, almost half of the nursing students reported positive attitudes, and more than half reported negative attitudes [26].

According to our results, there were some sociodemographic factors associated with knowledge of and attitudes toward LGBT care among nursing students. Sociodemographic factors such as age, religious affiliation, having an LGBT friend or relative, and being an undergraduate student without professional experience influenced attitudes toward LGBT individuals. Indeed, such factors have been identified as predictors for the formation of attitudes among the student population in previous studies in Greece [27]. It is worth noting that in this study, nursing students who declared themselves atheists showed more positive attitudes and a higher level of willingness to care for LGBT patients. This finding is in agreement with those of other studies [28,29] in which it seems that the religious element may determine the opinions and attitudes of students and health professionals. In fact, this effect is independent of the type of religion, as the phenomenon is observed in various religions (Catholicism, Orthodox Christianity, and Islam). In many deeply conservative societies, people’s religion, morals, and way of life have been and remain oriented towards heterosexual sexuality and condemn other sexual expressions [27]. Greek society is one of the most negative in Europe regarding homosexuality and marriages between homosexual couples, while the Greek Orthodox religion is closely linked to the ethics and practices of people, both in ancient times and in modern times. Also, this study revealed that being a friend with or closely knowing an LGBT person was associated with more positive attitudes and an increased level of knowledge on LGBT patients’ health issues. Similar results are mentioned in other studies [7,18,30]. It could be argued that these students are likely to be more aware of LGBT given their familiarity with the LGBT environment and their lives. On the contrary, those who stated that they do not socialize with LGBT individuals presented less-positive perceptions toward them. Probably because of their lack of socializing with people, they tend to reject or view with hesitation anyone who is “different” from them. In a study [31] among 1282 nursing educators and students in the United States, the authors mention that educators and students with more experience with LGBT patients had more positive attitudes.

The study has a few limitations worth noting. First, the NKALH survey was administered during university courses; therefore, the presence of other students may have affected the objectivity of the answers. Second, the study was conducted at two universities in Greece, and the findings may not be generalized to students at other universities in the country. Also, the percentage (19.5%) of individuals who declared themselves non-heterosexual is relatively large, and this may have influenced the level of positive perceptions Nonetheless, the major strength of this study is that it is the first study in Greece that aimed to translate and adapt this survey for Greek nursing students. Last, the findings of this study add to the literature on health professionals’ knowledge of and attitudes toward LGBT individuals.

## 5. Conclusions

The NKALH survey has good construct and convergent validity, reliability, and internal consistency for the Greek population. Thus, it is a reliable and valid tool for measuring the knowledge and attitudes of nursing students on LGBT health concerns. It is easily evaluated, allowing health professionals to integrate appropriate interventions into the care provided to LGBT individuals. In addition, it can be used not only among nursing students but also among nurses, thus making comparisons possible.

## Figures and Tables

**Table 1:** Students’ Sociodemographic Characteristics (N = 258).

		N	%
Gender	Male	44	17.1
	Female	210	81.4
	Intersex	1	0.4
	Other	3	1.2
Age (±SD)		23.5 (±7.8)	
Degree	Undergraduate student	233	90.3
	Nurse assistant to Bachelor of Science Nursing	25	9.7
Year of Study	1^st^	63	24.4
	2^nd^	54	20.9
	3^rd^	65	25.2
	4^th^	36	14.0
	5th and more	40	15.5
Sexual Orientation	Heterosexual	208	80.6
	Homosexual	11	4.3
	Bisexual	28	10.9
	Other	11	4.3
Nationality	Greek	242	93
	Other	17	7
Religion	Christian	204	78
	Atheist—not religious	44	17
	Muslims, Protestants	9	5
Yearly Income (€)	≤8.718	172	66.7
	8.719–17.435	56	21.7
	17.436–26.153	15	5.8
	26.154–34.871	8	3.1
	34.872–43.589	3	1.2
	≥43.590	4	1.6
Knowing an LGBT person	Yes	227	88.0
	No	31	12.0
Have you ever attended a class regarding LGBT care?	Yes	22	7.8
	No	236	91.2
Have you ever cared for an LGBT patient	Yes	65	24.8
	No	193	74.4

SD: Standard Deviation.

**Table 2:** Descriptive statistics for Nursing Students’ Knowledge and Attitudes of LGBT Health Concerns (NKALH) survey.

	Minimum	Maximum	Mean	Std. Deviation	Cronbach’s Alpha/KR-20
Knowledge of LGBT health concerns	0	30	16.49	5.755	0.805 *
Comfortable	2.22	4.89	3.95	0.47	0.701
Responsibility	1.67	5.00	3.82	0.64	0.728
Willingness to Care	1.80	5.50	4.34	0.73	0.816
Total Scale	2.21	4.82	4.041	0.53	0.783

* Kudar–Richardson 20 (KR-20).

**Table 3:** Correlation coefficient (r) between the factors of NKALH Survey.

	Comfortable	Responsibility	Willingness to Care	Total
Comfortable		0.554 **	0.620 **	0.801 **
Responsibility	0.554 **		0.665 **	0.870 **
Willingness for Care	0.620 **	0.665 **		0.907 **
Total	0.801 **	0.870 **	0.907 **	

** *p* < 0.01.

## Data Availability

Data are available from the corresponding author upon request.
